# Plasma Proprotein Convertase Subtilisin/kexin Type 9 (PCSK9) in the Acute Respiratory Distress Syndrome

**DOI:** 10.3389/fmed.2022.876046

**Published:** 2022-06-13

**Authors:** Thomas S. Metkus, Bo Soo Kim, Steven R. Jones, Seth S. Martin, Steven P. Schulman, Thorsten M. Leucker

**Affiliations:** ^1^Divisions of Cardiology and Cardiac Surgery, Departments of Medicine and Surgery, The Johns Hopkins University School of Medicine, Baltimore, MD, United States; ^2^Division of Pulmonary and Critical Care Medicine, Department of Medicine, The Johns Hopkins University School of Medicine, Baltimore, MD, United States; ^3^Division of Cardiology, Department of Medicine, The Johns Hopkins University School of Medicine, Baltimore, MD, United States

**Keywords:** PCSK9 (proprotein convertase subtilisin kexin type 9), ARDS (acute respiratory disease syndrome), critical illness ^*^mortality, ventilator free days, sepsis

## Abstract

**Background:**

Proprotein convertase subtilisin/kexin type 9 (PCSK9) is a serine protease that is a mediator of the immune response to sepsis. PCSK9 is also highly expressed in pneumocytes and pulmonary endothelial cells. We hypothesized that serum PCSK9 levels would be associated with death and ICU outcomes in patients with ARDS.

**Methods:**

Using data and plasma samples from the NIH BioLINCC data repository, we assembled a cohort of 1,577 patients with the acute respiratory distress syndrome (ARDS) enrolled in two previously completed clinical trials, EDEN and SAILS. We measured PCSK9 levels in plasma within 24 h of intubation using commercially available ELISA kits (R&D Systems). We assessed the association of PCSK9 with mortality using Cox proportional hazard models. We also assessed clinical factors associated with PCSK9 level and the association of PCSK9 with the number of days free of mechanical ventilation and days free of ICU care.

**Results:**

In 1,577 ARDS patients, median age was 53 years (IQR 42–65 years) and median APACHE III score 91 (72–111) connoting moderate critical illness. PCSK9 levels were 339.3 ng/mL (IQR 248.0–481.0). In multivariable models, race, cause of ARDS, body mass index, pre-existing liver disease, body temperature, sodium, white blood cell count and platelet count were associated with PCSK9 level. Presence of sepsis, use of vasopressors and ventilator parameters were not associated with PCSK9 level. PCSK9 levels were not associated with in-hospital mortality (HR per IQR 0.96, 95% CI 0.84–1.08, *P* = 0.47). Higher PCSK9 levels were associated with fewer ICU and ventilator free days.

**Conclusions:**

Plasma PCSK9 is not associated with mortality in ARDS, however higher PCSK9 levels are associated with secondary outcomes of fewer ICU free and ventilator free days. Clinical factors associated with PCSK9 in ARDS are largely unmodifiable. Further research to define the mechanism of this association is warranted.

## Introduction

The acute respiratory distress syndrome (ARDS) is a common cause of respiratory failure, associated with 1 in 10 ICU admissions (1) with mortality approaching 50% for the most severe cases (1) and novel strategies are needed for risk stratification and treatment. Proprotein convertase subtilisin/kexin type 9 (PCSK9) is a serine protease receiving increased attention for therapeutic role in cholesterol metabolism. PCSK9 has recently been implicated as a critical mediator of the immune response to sepsis, whereby PCSK9 inhibits hepatic clearance of lipopolysaccharide in the liver leading to increased inflammation and worse outcomes (2–4). PCSK9 is highly expressed in pneumocytes (alveolar epithelial cells type I and type II) and pulmonary endothelial cells (5) where its function is not known. This increasing body of evidence suggests that PCSK9 is expressed in extra-hepatic and extra-renal tissues including lung tissue and endothelial cells (4, 6, 7). Elevated levels of PSCK9 were associated with reduced clearance of endotoxin in sepsis and inhibiting PSCK9 attenuated inflammatory response to sepsis and improved survival in a mouse model (3). Similar findings of reduced inflammatory response to sepsis and improved outcomes were discovered among humans who had genetic loss-of-function of PCSK9 (3). PCSK9 serum levels were highly correlated with the development of subsequent multiple organ failure, which is a major mediator of mortality in ARDS (8). Thus, a conceptual model exists whereby PCSK9 is an inhibitor of clearance of circulating LPS in sepsis, and reduced PCSK9 activity is associated with an attenuated inflammatory response and improved outcome. Therefore, PCSK9 is a key mediator of the systemic inflammatory and endothelial response to critical illness, (3, 9) however its role in ARDS is not established.

We sought to characterize circulating PCSK9 as a novel biomarker of outcome in ARDS. We hypothesized that higher levels of circulating PCSK9 assessed by ELISA would be associated with increased mortality, fewer ventilator-free days, and more extrapulmonary organ dysfunction in patients with ARDS.

## Methods

### Study Population

Using data and plasma from the NIH BioLINCC data repository (10, 11), we constructed a cohort of 1,577 patients with ARDS enrolled in the previously completed EDEN trial (12) and SAILS trial (13). The EDEN trial enrolled patients from 44 hospitals with ARDS defined as hypoxemia with PaO2/fiO2 ratio < 300 and bilateral pulmonary infiltrates not clinically attributed to heart failure (12). Individuals were randomized within 48 h of ARDS onset to one of two enteral feeding strategies, and the endpoints of 60 day mortality, ventilator free days within 28 days of intubation and infectious complications were not different between groups (12). The SAILS trial included patients across 45 hospitals with ARDS defined identically to the EDEN trial (13). Additional inclusion criteria included suspected infection as a cause of ARDS and presence of criteria for the systemic inflammatory response syndrome (WBC > 12,000 or < 4000; or >10% band forms; or temperature >38°C or < 36°C) (13). We included individuals who were enrolled within 24–48 h of ARDS onset with available trial day 0 plasma.

### PCSK9 Measurement

We measured circulating PCSK9 levels in plasma using commercially available ELISA kits (R&D Systems). The limit of detection (LoD) of the assay is 0.6 ng/mL and coefficient of variation < 8%.

### Outcome and Covariates

The primary outcome was 90-day mortality from time of trial enrollment. The outcomes of ventilator free days and ICU free days were defined as the number of days out of 28 days that each patient was not receiving mechanical ventilation or not receiving ICU care, starting from trial enrollment. Models were adjusted for covariates of a priori interest based on our conceptual model including age, sex, trial and degree of critical illness.

### Statistical Analysis

Baseline characteristics are compared across quartiles of PCSK9 level using linear regression across quartiles for continuous variables and chi-squared tests for dichotomous variables. The distribution of PCSK9 was right skewed and therefore PCSK9 was log-transformed for use as the dependent variable in linear regression models. Univariable regression models were performed with clinical characteristics as the independent variable and natural log of PCSK9 as the dependent variable. Covariates were entered in the multivariable model if *P* < 0.1 in univariable analysis. Covariates were removed from the multivariable model if non-significant at a *P* < 0.05 level and assessed as a group using the Wald test. The final covariates in the model were verified by comparing the model to models generated using both automatic forward and backward stepwise regression. Model diagnostics include assessment of the variance inflation factor, inspection of the residuals vs. predicted values plot and inspection of the residuals for normal distribution using Q-Q plots.

For the survival analysis, PCSK9 was rescaled by a factor representing the inter-quartile range. We performed Cox proportional hazard modeling adjusting for factors determined a priori and plotted Kaplan-Meier survival curves. The proportional hazard assumption was assessed and satisfied. To determine the association of PCSK9 with the count of ICU free days and ventilator free days, we first fitted adjusted models using Poisson regression, however the model was overdispersed based on goodness-of-fit test. Due to the fact that over 24% of patients had 0 ICU free and ventilator free days, we proceeded to fit zero-inflated Poisson and negative binomial models. The models were compared using Akaike information criteria and the zero inflated negative binomial model was selected. All analyses were performed using Stata 14.0. A two-tailed *p* value < 0.05 was considered statistically significant.

## Results

### PCSK9 in ARDS

**Figure 1:**
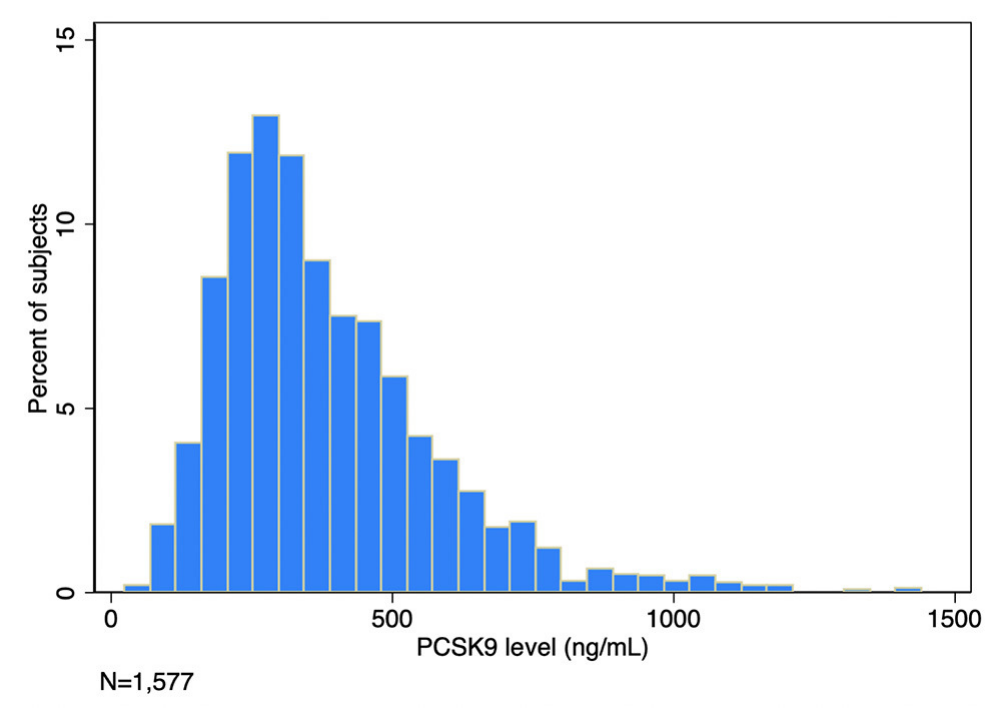
Circulating plasma PCSK9 in 1,577 subjects with ARDS.

In 1,577 ARDS patients, median age was 53 years (IQR 42–65 years), 76% had pneumonia or aspiration as the cause of ARDS, 52% required vasopressors, and the median APACHE III score was 91 (72–111), connoting moderate critical illness (Table 1). Median PCSK9 level in the ARDS cohort was 339.3 (IQR 247.8–481.0) ng/mL with right-skewed distribution as shown in Figure 1. Patients with greater PCSK9 levels had higher weight and body mass index, lower white blood cell counts and higher temperature and glucose levels. Patients with lower PCSK9 levels had a greater frequency of chronic liver disease and cirrhosis (Table 1).

**Table 1:** Characteristics of study participants by quartile of PCSK9 level.

		**Quartile of PCSK−9 level (Range, ng/mL)**				
**Characteristics**	**Overall**	**First (25.2–247.8)**	**Second (248–339.2)**	**Third (339.3–480.6)**	**Fourth (481.0–1440.6)**	**p**
Number	1577	394	393	395	395	
PCSK9 level (ng/mL)	339.3 (248.0–481.0)	197.4 (163.5–226.8)	293.2 (269.8–312.5)	405.8 (370.4–441.6)	602.1 (528.7–718.6)	
Age (years)	53 (42–65)	54 (43–64)	52 (42–64)	53 (41–65)	54 (43–64)	0.98
Female sex [*N* (%)]	784 (49.7)	199 (50.5)	202 (51.4)	198 (50.1)	185 (46.8)	0.6
Race [*N* (%)]						0.09
White	1216 (77.1)	296 (75.1)	300 (76.3)	315 (79.8)	305 (77.2)	
African–American	239 (15.2)	75 (19.0)	59 (15.0)	53 (13.4)	52 (13.2)	
Other	122 (7.7)	23 (5.8)	34 (8.7)	27 (6.8)	38 (9.6)	
Medical ICU [*N* (%)]	990 (63.8)	267 (67.8)	241 (61.3)	254 (64.3)	228 (57.7)	0.03
Cause of ARDS [*N* (%)]						0.33
Sepsis	256 (16.3)	76 (19.4)	56 (14.3)	63 (16.0)	61 (15.4)	
Trauma	36 (2.3)	6 (1.5)	9 (2.3)	5 (1.3)	16 (4.1)	
Transfusion	19 (1.2)	5 (1.3)	4 (1.0)	5 (1.3)	5 (1.3)	
Aspiration	129 (8.2)	35 (8.9)	26 (6.7)	35 (8.9)	33 (8.4)	
Pneumonia	1069 (68)	258 (65.8)	280 (71.6)	271 (68.8)	260 (65.8)	
Other	63 (4.0)	12 (3.1)	16 (4.1)	15 (3.8)	20 (5.1)	
Height (cm)	168 (162.5–176.5)	167.6 (160–177)	170 (162.3–175.7)	168 (162.6–175.3)	170 (162.6–177.8)	0.28
Weight (kg)	82 (68–100)	78 (67–95)	85 (69–100)	83 (68–100)	84 (69–103)	0.002
BMI (kg/m2)	28.6 (23.8–34.7)	27.8 (23.1–33.1)	28.9 (23.8–35.1)	28.9 (23.9–35.2)	29.0 (24.2–35.9)	0.006
APACHE III score	91 (72–111)	93 (73–114)	90 (74–109)	84 (68–106)	93 (72–114)	0.51
Comorbidities [*N* (%)]						
Chronic dialysis	44 (2.8)	17 (4.3)	7 (1.8)	8 (2.0)	12 (3.0)	0.12
AIDS	37 (2.4)	8 (2.0)	8 (2.0)	9 (2.3)	12 (3.0)	0.76
Leukemia	59 (3.7)	10 (2.5)	19 (4.8)	13 (3.2)	17 (4.3)	0.33
Lymphoma	18 (1.1)	8 (2.0)	4 (1.0)	1 (0.3)	5 (1.3)	0.13
Solid tumor	41 (2.6)	15 (3.8)	12 (3.1)	6 (1.5)	8 (2.0)	0.18
Immunodeficiency	202 (12.8)	47 (11.9)	61 (15.5)	37 (9.3)	57 (14.4)	0.05
Hepatic disease	17 (1.1)	‘5 (3.8)	0 (0)	1 (0.3)	1 (0.3)	<0.001
Cirrhosis	74 (4.7)	49 (12.5)	13 (3.3)	7 (1.8)	5 (1.3)	<0.001
Diabetes	408 (25.9)	101 (25.7)	93 (23.7)	93 (23.5)	121 (30.6)	0.08
Hypertension	742 (47.1)	176 (44.8)	182 (46.3)	181 (45.8)	203 (51.4)	0.25
Prior MI	80 (5.1)	19 (4.8)	18 (4.6)	24 (6.1)	19 (4.8)	0.77
Prior heart failure	101 (6.4)	24 (6.1)	26 (6.6)	24 (6.1)	27 (6.8)	0.96
Peripheral vascular disease	74 (4.7)	23 (5.8)	23 (5.9)	10 (2.5)	18 (4.6)	0.09
Stroke/TIA	54 (3.4)	14 (3.6)	13 (3.3)	14 (3.5)	13 (3.3)	0.99
Chronic pulmonary disease	234 (14.9)	59 (15.0)	70 (17.8)	56 (14.1)	49 (12.4)	0.19
Laboratory evaluation						
WBC, high (cells/mm3)	12.7 (8.1–18.2)	13.7 (8.3–18.9)	14.0 (9.5–19.4)	12.6 (8.4–18.9)	10.9 (7.1–15.7)	0.02
Platelets (cells/mm3)	172 (107–246)	171 (96–243)	186 (108–271)	182 (172–244)	151 (110–214)	0.12
Sodium, low (mEQ/L)	138 (134–141)	138 (134–141)	138 (134–140)	137 (134–140)	138 (134–142)	0.03
Potassium, high (mEQ/L)	4.2 (3.8–4.6)	4.1 (3.7–4.5)	4.2 (3.9–4.6)	4.1 (3.7–4.6)	4.2 (3.8–4.6)	0.85
Creatinine, high (mg/dL)	1.2 (0.8–2.0)	1.3 (0.8–2.0)	1.2 (0.8–1.9)	1.1 (0.8–1.7)	1.2 (0.8–2.1)	0.82
Glucose, high (mg/dL)	145 (116–189)	140 (109–182)	144 (115–184)	142 (114–186)	155 (124–207)	0.001
Bicarbonate (mEQ/L)	22 (18–25)	21 (18–25)	22 (18–24)	22 (19–26)	22 (18–25)	0.15
Sepsis [*N* (%)]						0.53
Primary diagnosis	260 (16.5)	77 (19.5)	58 (14.8)	64 (16.2)	61 (15.4)	
Secondary diagnosis	777 (49.3)	188 (47.7)	203 (51.7)	197 (49.9)	189 (47.9)	
Vasopressor use [*N* (%)]	815 (51.7)	202 (51.3)	208 (52.9)	209 (52.9)	196 (49.6)	0.76
Temperature, high (degrees C)	38.1 (37.4–38.9)	37.9 (37.2–38.6)	38.1 (37.3–38.9)	38.2 (37.5–38.9)	38.3 (37.6–39.1)	<0.001
HR, high (beats per minute)	116 (102–131)	115 (100–131)	117 (103–130)	114 (100–129)	120 (103–135)	0.041
SBP, low (mmHg)	85 (76–94)	84 (77–92)	84 (76–94)	85 (77–94)	86 (77–95)	0.24
Urine out, day 0 (mL)	1333 (771–2155)	1210 (605–1885)	1400 (797–2125)	1374 (811–2305)	1408 (815–2217)	0.2
Fluid balance, day 0 (mL)	1932 (384–3880)	1888 (289–4094)	2009 (412–3967)	1934 (132–3485)	1913 (603–3831)	0.84
pH	7.37 (7.31–7.42)	7.36 (7.30–7.41)	7.37 (7.33–7.42)	7.37 (7.32–7.42)	7.37 (7.31–7.42)	0.42
PaO2 (mmHg)	84 (70–105)	83 (70–107)	86 (73–108)	84 (68–108)	81 (69–100)	0.23
PaCO2 (mmHg)	38 (33–45)	39 (34–45)	38 (34–45)	40 (34–46)	37 (32–44)	0.83
P/F ratio	150 (109–202)	150 (114–205)	153 (110–203)	152 (112–203)	143 (101–196)	0.16
Death [N, (%)]	386 (24.5)	121 (30.7)	81 (20.6)	79 (20.0)	105 (26.6)	0.001
ICU free days	18 (1–23)	18 (0–23)	20 (8–23)	20 (4–24)	15 (0–22)	0.19
Ventilator free days	20 (0–24)	19 (0–24)	21 (0–24)	22 (0–25)	17 (0–24)	0.65

### Factors Associated With PCSK9 Levels

Table 2 displays the univariable regression model for clinical factors associated with PCSK9 levels in ARDS. Race, weight, cause of ARDS, as well as comorbidities of liver disease and hypertension and glucose levels were associated with PSCK9 levels. In multivariable models, shown in Table 3, cause of ARDS, race, BMI and medical compared to surgical admission, chronic liver disease, WBC, glucose and sodium levels and temperature were associated with PCSK9 levels. Degree of critical illness, presence of sepsis or shock, and more severe lung disease were not associated with PCSK9 levels. The R2 for the multivariable model was 0.108.

**Table 2:** Univariable regression table for factors associated with PCSK9 level in ARDS.

	**Univariable model**	
**Characteristics**	**Beta (SE)**	**P**
Age (years)	0.0071 (0.00077)	0.78
Female sex [*N* (%)]	−0.027 (0.025)	0.28
Race [*N* (%)]		
White	Ref	Ref
African–American	−0.057 (0.035)	0.025
Other	0.030 (0.047)	0.23
Medical ICU [*N* (%)]	−0.074 (0.025)	0.003
Cause of ARDS [*N* (%)]		
Sepsis	Ref	Ref
Trauma	0.070 (0.089)	0.008
Transfusion	0.010 (0.12)	0.69
Aspiration	0.035 (0.053)	0.24
Pneumonia	0.055 (0.034)	0.088
Other	0.049 (0.070)	0.073
Shock	−0.051 (0.25)	0.041
Height (cm)	0.013 (0.001)	0.61
Weight (kg)	0.077 (0.00046)	0.002
BMI (kg/m2)	0.077 (0.0014)	0.002
APACHE III score	−0.026 (0.00046)	0.31
Comorbidities [*N* (%)]		
Chronic dialysis	−0.0071 (0.076)	0.78
AIDS	0.029 (0.082)	0.24
Leukemia	0.016 (0.066)	0.53
Lymphoma	−0.031 (0.12)	0.21
Solid tumor	−0.049 (0.078)	0.052
Immunodeficiency	0.0099 (0.037)	0.7
Hepatic disease	−0.013 (0.12)	<0.001
Cirrhosis	0.024 (0.057)	<0.001
Diabetes	0.040 (0.028)	0.12
Hypertension	0.054 (0.025)	0.033
Prior MI	0.028 (0.057)	0.27
Prior heart failure	0.023 (0.051)	0.36
Peripheral vascular disease	−0.034 (0.059)	0.18
Stroke/TIA	0.021 (0.069)	0.4
Chronic pulmonary disease	−0.019 (0.035)	0.46
Laboratory evaluation		
WBC, high (cells/mm3)	−0.062 (0.0001)	0.01
Platelets (cells/mm3)	0.029 (0.00011)	0.25
Sodium, low (mEQ/L)	0.050 (0.0023)	0.048
Potassium, high (mEQ/L)	−0.00034 (0.018)	0.99
Creatinine, high (mg/dL)	0.016 (0.0088)	0.52
Glucose, high (mg/dL)	0.088 (0.00015)	<0.0001
Bicarbonate (mEQ/L)	0.0532 (0.0024)	0.035
Sepsis [N (%)]		
Primary diagnosis	−0.070 (0.37)	0.01
Secondary diagnosis	−0.043 (0.028)	0.12
Vasopressor use [N (%)]	0.025 (0.025)	0.29
Temperature, high (degrees C)	0.17 (0.012)	<0.001
HR, high (beats per minute)	0.042 (0.00054)	0.092
SBP, low (mmHg)	0.046 (0.00079)	0.07
Urine out, day 0 (mL)	0.040 (0.00001)	0.12
Fluid balance, day 0 (mL)	−0.015 (0.0001)	0.54
pH	0.052 (0.15)	0.042
PaO2 (mmHg)	−0.026 (0.00035)	0.3
PaCO2 (mmHg)	−0.0022 (0.0012)	0.93
P/F ratio	−0.031 (0.00017)	0.23

**Table 3:** Multivariable regression table for factors associated with PCSK9 level in ARDS.

	**Multivariable model**	
**Characteristics**	**Beta (SE)**	**P**
White	Ref	Ref
African–American	−0.074 (0.034)	0.002
Other	0.043 (0.045)	0.075
Medical ICU [*N* (%)]	−0.050 (0.025)	0.042
Cause of ARDS [*N* (%)]		
Sepsis	Ref	Ref
Trauma	0.056 (0.085)	0.03
Transfusion	0.024 (0.11)	0.33
Aspiration	0.024 (0.051)	0.4
Pneumonia	0.068 (0.033)	0.03
Other	0.053 (0.067)	0.04
BMI (kg/m2)	0.062 (0.0013)	0.01
Comorbidities [*N* (%)]		
Hepatic disease	0.056 (0.13)	0.034
Cirrhosis	−0.20 (0.061)	<0.001
Laboratory evaluation		
WBC, high (cells/mm3)	−0.050 (0.00001)	0.039
Sodium, low (mEQ/L)	0.066 (0.0022)	0.007
Glucose, high (mg/dL)	0.089 (0.00014)	<0.0001
Temperature, high (degrees C)	0.15 (0.011)	<0.001

### Association of PCSK9 With Outcome

**Figure 2:**
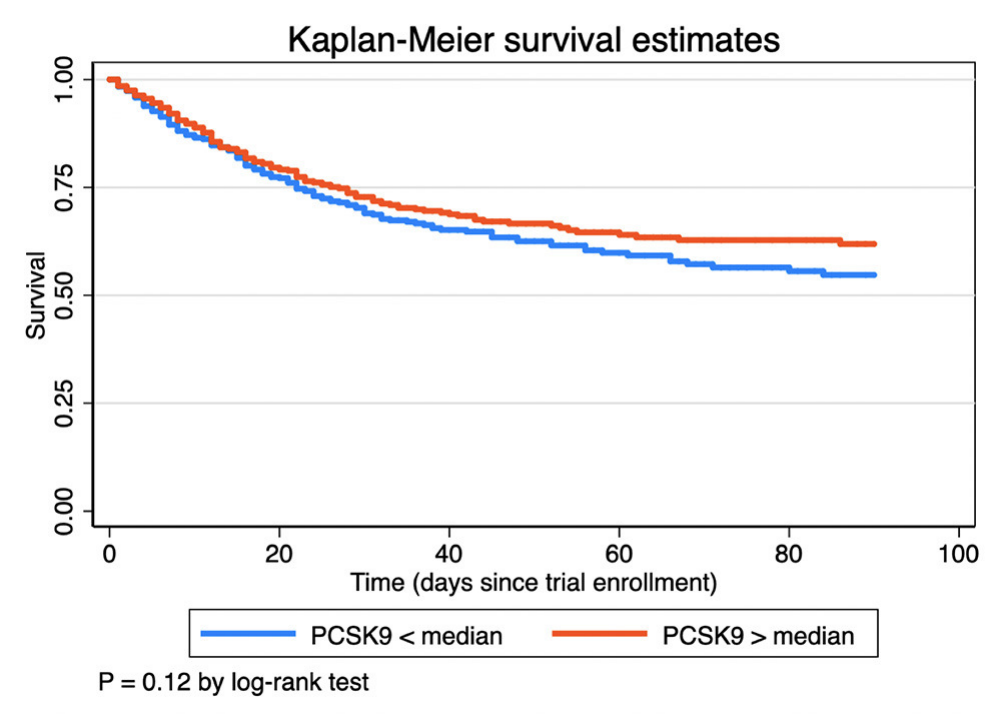
Kaplan-Meier curves for 90 day mortality as a function of PCSK9 greater or less than the median value.

Survival curves for PCSK9 greater vs. less than the median value are shown in Figure 2, demonstrating no association of median PCSK9 level at day 0 with mortality. PCSK9 was not associated with mortality when considered as a continuous variable in univariable (HR 0.99 95% CI 0.86–1.14, *P* = 0.91) or adjusted (HR 0.93 95% CI 0.81–1.06, *P* = 0.26) models as shown in Table 4. Greater PCSK9 levels were associated with fewer ICU free days (IRR 0.96 per IQR of PCSK9, 95% CI 0.93–0.99, *p* < 0.004) and ventilator-free days (IRR 0.96 per IQR of PCSK9, 95% CI 0.94–0.98, *p* = 0.001), Table 4.

**Table 4:** Cox model adjusted for age, sex, trial and severity of critical illness. Models of the association of PCSK9 level with the secondary outcomes of ICU free and ventilator free days.

	**Hazard ratio or Incidence rate ratio (95% CI) per IQR of PCSK9**	**P**
90 day mortality	0.96 (0.84–1.08)	0.47
ICU free days	0.96 (0.93–0.99)	0.004
Ventilator free days	0.96 (0.94–0.98)	0.001

*Models adjusted for age, sex, trial and APACHE III score*.

## Discussion

In this retrospective cohort study characterizing circulating serum PCSK9 levels in 1,577 patients with ARDS, we report several findings. First, circulating PCSK9 is detectable in ARDS with right-skewed distribution. Second, factors associated with PCSK9 levels in ARDS are largely unmodifiable and responsible for only a minority of the variation in levels. Finally, PCSK9 was not associated with mortality and may be associated with ICU-free and ventilator-free days. These data characterize PCSK9 as a biomarker in ARDS that was not associated with mortality, contrary to our hypothesis.

### PCSK9 Levels in Critical Illness

Our results suggest that circulating PCSK9 is detectable in critical illness. Ridker et al. report baseline PCSK9 levels of approximately 300–305 ng/mL among previously healthy individuals who did and did not subsequently suffer a cardiovascular event (14). Pastori et al. reported levels of approximately 1,500 pg/mL in a cohort of patients with atrial fibrillation (15) and Laugsand et al. reported levels of approximately 150 ng/mL in the general population in Norway (16), both numerically lower than the levels observed in our cohort. Assays for PCSK9 are not standardized, however, which precludes direct comparison.

### Specific Molecular Pathways Involved Between PCSK9 and Lung Injury

Most of the current PCSK9 literature centers around the development and progression of atherosclerotic cardiovascular disease. However, experimental evidence suggests that PCSK9 inhibition decreases inflammation via inhibiting the TLR4/NF-κB signaling pathway without having a significant impact on plasma cholesterol level. The TLR4/NF-κB signaling pathway has been found to be one of the main affected pathways mediating the PCSK9-induced increase in pro-inflammatory cytokines (17). Furthermore, data from a sepsis/ARDS rodent model suggests that inhibition of TLR4 signaling pathway may relieve sepsis-associated ARDS in through regulating macrophage activation and the inflammatory response (18). Furthermore, although PCSK9 is detected in lung tissue and may be involved in clearance of pathogenic phospholipids from the alveolar space after lung injury (19, 20), the precise mechanisms of PCSK9 in pulmonary epithelial and endothelial cells remains unknown at this point.

An upcoming study may shed more light on the importance of PCSK9 and associated molecular pathways in patients at risk for acute lung injury: IMPACT-SIRIO 5 study (NCT04941105). The IMPACT-SIRIO 5 is a randomized, double-blind, phase III clinical trial evaluating the safety and efficacy of PCSK9 inhibition on clinical outcome (need for intubation, death of any cause, and changes in IL-6 concentrations) in patients during the acute inflammatory stage of COVID-19 (21).

### Factors Associated With PCSK9 Levels

Despite observing elevated PCSK9 levels, we found that clinical factors associated with critical illness explained only a minority of variation in PCSK9 levels. The negative association of PCSK9 with liver disease is intuitive given the role of PCSK9 with hepatic cholesterol metabolism- PCSK9 is intimately involved in cholesterol metabolism, by binding to the EGF-A domain of the low-density lipoprotein receptor (LDLR) and targeting it for lysosomal-mediated degradation (22). Similarly, the association of WBC and temperature with PCSK9 are consistent with the purported role of PCSK9 with the inflammatory cascade: elevated levels of PSCK9 were associated with reduced clearance of endotoxin in sepsis and inhibiting PSCK9 attenuated inflammatory response to sepsis and improved survival in a mouse model (3). Similar findings of reduced inflammatory response to sepsis and improved outcomes were discovered among humans who had genetic loss-of-function of PCSK9 (3). Other studies have found positive correlations between PCSK9 and inflammatory markers (23). PCSK9 serum levels were highly correlated with the development of subsequent multiple organ failure which is a major mediator of mortality in ARDS (8).

Despite these prior studies, our results suggest that factors associated with PCSK9 are largely unmodifiable, and it is notable that we found no association between degree of critical illness, presence of sepsis or severity of lung disease and PCSK9 levels. This is consistent with a study that observed no association of PCSK9 with severity of illness in patients with bacterial infection in the ICU (24). That observed variables account for only approximately 10% of the variation in PCSK9 levels in this ARDS cohort suggest that deeper understanding of the mechanism underlying circulation of PCSK9 in ARDS is needed.

### PCSK9 and Outcome

PCSK9 was not associated with mortality in ARDS, contrary to our a priori hypothesis. In prior studies PCSK9 was associated with outcome in both human and murine models of septic shock (3, 8). PCSK9 was also associated with outcome in some (14, 25, 26) but not all (16) epidemiology studies in cardiac patients. That we observe association of higher PCSK9 with fewer ICU and ventilator free days but not mortality is consistent with prior literature that the association between circulating PCSK9 levels and clinical outcomes are at best mixed and unclear, perhaps due to the heterogenous causes of death in ARDS (27). A recent study suggested that neither genetic variants in PCSK9 nor predicted PCSK9 expression were associated with sepsis, cardiovascular events or death (28). Rannniko et al. found that lower levels of PCSK9 were associated with greater risk in the ICU (23) and inhibiting PCSK9 did not improve outcome of mice challenged with a septic stimulus (29). Overall, our results do not support the utility of PCSK9 as a biomarker of outcome in ARDS.

### Limitations

Limitations of our study include retrospective, observational design. Thus, we describe associations rather than causal effects. Not all patients in the studies comprising the cohort had available plasma for analysis and it is not known if plasma on trial day 0 is representative of the trajectory of critical illness. The specific cause of death was not available in the parent studies, thus all-cause mortality rather than cause-specific mortality is reported. The assays for assessment of PCSK9 are not standardized in the literature, hence, these findings are exploratory. Finally, these trials were conducted in the past and it is possible that the epidemiology of critical illness and ARDS has changed in the past decade.

## Conclusion

Circulating PCSK9 is elevated in ARDS and factors associated with PCSK9 levels in ARDS are largely unmodifiable and responsible for only a minority of the variation in levels. PCSK9 levels were not associated with mortality and may be associated with ICU-free and ventilator-free days. These data characterize PCSK9 as a biomarker in ARDS. Mechanistic data as to the role of PCSK9 in critical illness is needed.

## Data Availability Statement

The raw data supporting the conclusions of this article will be made available by the authors, without undue reservation.

## Ethics Statement

The EDEN and SAILS studies were approved by all participating Institutional Review Boards. The Johns Hopkins Hospital Institutional Review Board exempted our study (IRB #00173035), given that all study data was publicly available from the BIOLINCC program (10, 11) and that the data sets contained no protected health information.

## Author Contributions

TM designed the study, performed data analysis and drafted the manuscript. BK designed the study and provided critical edits to the manuscript. SJ, SM, and SS provided critical edits to the manuscript. TL designed the study and provided critical edits to the manuscript. All authors contributed to the article and approved the submitted version.

## Funding

This work was supported by a Johns Hopkins Division of Cardiology Magic that Matters award to TM and TL.

## Conflict of Interest

The authors declare that the research was conducted in the absence of any commercial or financial relationships that could be construed as a potential conflict of interest.

## Publisher's Note

All claims expressed in this article are solely those of the authors and do not necessarily represent those of their affiliated organizations, or those of the publisher, the editors and the reviewers. Any product that may be evaluated in this article, or claim that may be made by its manufacturer, is not guaranteed or endorsed by the publisher.
